# Activation of ADAM17 by IL-15 Limits Human NK Cell Proliferation

**DOI:** 10.3389/fimmu.2021.711621

**Published:** 2021-07-22

**Authors:** Hemant K. Mishra, Kate J. Dixon, Nabendu Pore, Martin Felices, Jeffrey S. Miller, Bruce Walcheck

**Affiliations:** ^1^ Department of Veterinary and Biomedical Sciences, University of Minnesota, St. Paul, MN, United States; ^2^ Early Oncology Clinical Science, AstraZeneca, Gaithersburg, MD, United States; ^3^ Department of Medicine, Division of Hematology, Oncology, and Transplantation, University of Minnesota, Minneapolis, MN, United States

**Keywords:** natural killer cell, ADAM17 (a disintegrin and metalloprotease 17), CD62L, proliferation, IL-15

## Abstract

Natural killer (NK) cells are innate cytotoxic lymphocytes that can recognize assorted determinants on tumor cells and rapidly kill these cells. Due to their anti-tumor effector functions and potential for allogeneic use, various NK cell platforms are being examined for adoptive cell therapies. However, their limited *in vivo* persistence is a current challenge. Cytokine-mediated activation of these cells is under extensive investigation and interleukin-15 (IL-15) is a particular focus since it drives their activation and proliferation. IL-15 efficacy though is limited in part by its induction of regulatory checkpoints. A disintegrin and metalloproteinase-17 (ADAM17) is broadly expressed by leukocytes, including NK cells, and it plays a central role in cleaving cell surface receptors, a process that regulates cell activation and cell-cell interactions. We report that ADAM17 blockade with a monoclonal antibody markedly increased human NK cell proliferation by IL-15 both *in vitro* and in a xenograft mouse model. Blocking ADAM17 resulted in a significant increase in surface levels of the homing receptor CD62L on proliferating NK cells. We show that NK cell proliferation *in vivo* by IL-15 and the augmentation of this process upon blocking ADAM17 are dependent on CD62L. Hence, our findings reveal for the first time that ADAM17 activation in NK cells by IL-15 limits their proliferation, presumably functioning as a feedback system, and that its substrate CD62L has a key role in this process *in vivo*. ADAM17 blockade in combination with IL-15 may provide a new approach to improve NK cell persistence and function in cancer patients.

## Introduction

NK cells interrogate cells in the body for infection and transformation and eliminate these cells by rapidly induced effector activities, including a potent cytolytic process (1). The importance of these innate lymphocytes in cancer immunosurveillance is highlighted in NK cell deficient or depleted animal models where their absence results in failure to reject tumors (2). In addition, NK cell functional abnormalities in humans correlate with an increased risk of certain types of cancer (3). Human peripheral blood NK cells are identified as CD56^+^ CD3^−^, and their effector activities are rapidly induced by numerous germline-encoded receptors that respond to ligands upregulated and downregulated on tumor cells as well as attached antibodies (1). In consideration of this and their potential for allogeneic use, various NK cell platforms are being evaluated for adoptive cell therapies to treat hematologic malignancies and solid tumors (4, 5).

A limitation of adoptively transferred primary NK cells is their relatively short life span (6). IL-15 is critical for NK cell development, proliferation, and persistence (7). The cytokine binds to a heterotrimeric receptor that consists of the common gamma chain (γc) subunit, the beta chain (βc) subunit (IL-2/IL-15R) shared with the IL-2 receptor, and the IL-15Rα subunit (8). Recombinant human (rh) IL-15, derived IL-15 agonists, and human IL-15 transgene expression have been examined in immunocompromised mouse models and shown to promote the proliferation and effector functions of adoptively transferred human NK cells (9–12). IL-15 is also being examined in various clinical trials (13), and Cooley et al., have recently reported the results of a first-in-human trial of rhIL-15 and allogeneic NK cell therapy for advanced acute myeloid leukemia (14). Challenges for IL-15 immunotherapy, however, include the inhibitory actions of immunological checkpoints that it induces, and thus there is a considerable emphasis on identifying new mechanisms of action that improve the functionality of IL-15 therapy in cancer patients (13).

ADAM17 is a membrane-associated protease that mediates the “cleavage or shedding” of various cell surface proteins (15–17). This process can rapidly reduce the density of various receptors on leukocytes and regulates their activation as well as cell-cell interactions (18). ADAM17 is constitutively expressed by all human peripheral NK cells and its proteolytic activity is rapidly induced by assorted stimuli (19–22), including IL-15 (23). We show that ADAM17 activity regulates IL-15-mediated NK cell proliferation *in vitro* and *in vivo*, and that the homing receptor CD62L is a substrate involved in this process. The impact of these findings is that blocking ADAM17 function in combination with IL-15 stimulation may provide a new therapeutic approach to increase NK cell proliferation and their anti-tumor function in patients.

## Materials and Methods

### Reagents

The anti-ADAM17 monoclonal antibody (mAb) MEDI3622 (human IgG1) has been previously described (22, 24). All commercially available mAbs are listed in **Table 1**. Recombinant human (rh) IL-15 was obtained from the Biological Resources Branch, NCI, NIH and from R&D Systems (Minneapolis, MN).

**Table 1:** Description of the commercial antibodies used in this study.

Antigen	Clone	Catalogue #	Company
CD56	HCD56	318318	BioLegend, San Diego, CA
CD3	HIT3a	300440	BioLegend
CD16	3G8	302038	BioLegend
CD336/NKp44	P44-8	325108	BioLegend
CD335/NKp46	9E2	331914	BioLegend
CD159a/NKG2A	Z199	A60797	Beckman Coulter, Brea, CA
CD314/NKG2D	1D11	320806	BioLegend
CD158a/KIR2DL1	HP-MA4	339504	BioLegend
CD158b1/KIR2DL2/L3	DX27	312612	BioLegend
CD158e1/KIR3DL1	DX9	312714	BioLegend
CD45	HI30	304044	BioLegend
CD62L/L-selectin	DREG56	304810	Biolegend
CD62L/L-selectin	DREG200	HB302	ATCC, Manassas, VA
CD156b/ADAM17	D1(A12)	AB00611-10.0	Absolute Antibody Limited, Oxford, UK
*In vivo* grade isotype control, human IgG1	CB1	C0001	Crown Bioscience, San Diego, CA
*In vivo* grade isotype control, mouse IgG1	CB5	C0005	Crown Bioscience

### NK Cell Isolation

Fresh human peripheral blood leukocytes from plateletpheresis were obtained from Innovative Blood Resources (St. Paul, MN). PBMCs were further enriched by Ficoll-Paque Plus (GE Healthcare Bio-Sciences AB, Uppsala, Sweden) gradient and then NK cells were purified by negative depletion using isolation kits from StemCell Technologies (Cambridge, MA) or Miltenyi Biotec (Auburn, CA), as per the manufacturer’s instructions, with > 95% viability and ≥ 90% enrichment of CD56^+^ CD3^−^ lymphocytes. Viable cell counting was performed using a Countess II automated cell counter (Life Technologies Corporation, Bothell, WA).

### *In Vitro* NK Cell Proliferation

Enriched NK cells were labeled with CellTrace Violet Cell Proliferation Dye (ThermoFisher Scientific) per manufacturer’s instructions and incubated for 7 days in media containing or lacking rhIL-15 (R&D Systems), as we have previously described (25). In some experiments, MEDI3622, DREG200, and/or control IgG1 at 5μg/ml each were added to the assay, as indicated. An expansion index was calculated using FlowJo software (FlowJo, Ashland, OR) and represents the fold expansion of the overall culture based on CellTrace Violet dilution.

### Human NK Cell Adoptive Transfer

The xenogeneic adoptive transfer model was performed as we have previously described (10). NOD-*scid* IL2Rgamma^null^ (NSG) mice (stock number is 005557 from Jackson Laboratory, Bar Harbor, ME) were housed in a specific pathogen-free facility. Weight matched (26-30g) female mice were subjected to whole-body preconditioning irradiation (225 cGy using an X RAD 320, Precision X-ray, North Branford, CT, USA) for consistent NK cell engraftment. Freshly, enriched human NK cells underwent initial overnight incubation in B0 media [DMEM, Ham’s F12 with 10% human AB serum, Pen/Strep (1%), 2-ME (20 μm), ethanolamine (50 μm), ascorbic Acid (10 μg/ml) and sodium selenite (1.6 ng/ml)] containing 2.5 ng/ml rhIL-15 (NCI), and 4x10^6^ cells were injected *via* tail vein in each mouse. Mice were also administered rhIL-15 (NCI) *ip* at dose of 5 μg. The indicated mAbs were *ip* administered at a dose of 10 mg/kg. A schematic of the treatment schema is provided in **Figure 2A**. Blood was collected *via* retro-orbital route in heparin. Absolute counting of human NK cells in the peripheral blood was performed on a flow cytometer using a bead counting method (AccuCheck, Thermo Scientific, Waltham, MA, USA) according to the manufacturer’s instructions.

### Flow Cytometric Analyses

NK cells were stained with the indicated antibodies and examined by flow cytometry, as previously described (22). For controls, fluorescence minus one was used as well as appropriate isotype-matched antibodies since NK cells express Fc receptors. An FSC-A/SSC-A plot was used to set an electronic gate on leukocyte populations, and an FSC-A/FSC-H plot was used to set an electronic gate on single cells. To distinguish live vs. dead cells, 7AAD was used as per the manufacturer’s instructions (Biolegend, San Diego, CA).

### Statistical Analysis

Data were analyzed using Prism Graph Pad software. Student’s t-test or one-way ANOVA with multiple comparisons were used to determine statistical significance among groups.

## Results

### ADAM17 Blockade Increases NK Cell Proliferation by IL-15

**Figure 1:**
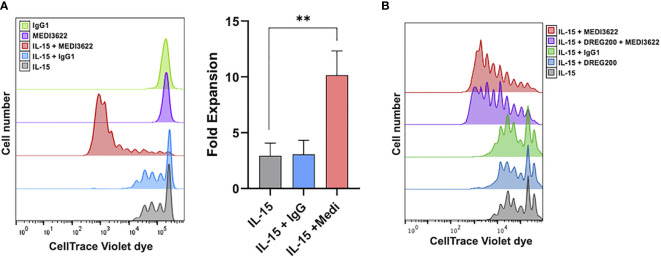
ADAM17 blockade enhances human NK cell proliferation by IL-15 *in vitro*. **(A)** Enriched NK cells were labeled with CellTrace Violet dye and placed in culture for 7 days with rhIL-15 (10ng/ml) and/or MEDI3622 (5μg/ml) and/or control human IgG1 (5μg/ml), as indicated. Cells were then harvested and examined for CellTrace dye dilution by flow cytometry. Data are representative of 3 independent experiments using leukocytes from separate donors. An expansion index was calculated as described in the Methods and is the fold expansion of the overall culture for each condition based on dye dilution. Data are means ± SD of three independent experiments using separate donors. Statistical significance is indicated as **p < 0.01. Statistics were calculated using one-way ANOVA. **(B)** Human PBMCs were labeled with CellTrace Violet dye and placed in culture for 7 days with rhIL-15 (10ng/ml), MEDI3622 (5μg/ml), control human IgG1 (5μg/ml), and/or DREG200 (5μg/ml). Cells were then harvested and examined for CellTrace dye dilution by flow cytometry. Data are representative of 3 independent experiments using leukocytes from separate donors.

We have previously reported that IL-15 stimulation of human NK cells activates ADAM17 in short-term experiments (≤ 24 hours) (23). We examined here if the sheddase had a role in NK cell proliferation during prolonged IL-15 stimulation. ADAM17 was blocked using MEDI3622, a human IgG1 mAb that is well characterized for its inhibitory activity *in vitro* and *in vivo* (24, 26, 27), and also blocks ADAM17 activity in human NK cells (22). Enriched NK cells were labeled with CellTrace dye, cultured for seven days in media containing or lacking rhIL-15 and/or MEDI3622, and then the cells were assessed for dye dilution. In the presence of rhIL-15, NK cells demonstrated increased dye dilution and thus proliferation (**Figure 1A**). We found that in the presence of MEDI3622, but not an isotype-matched control antibody, NK cell proliferation was greatly augmented (**Figure 1A**). NK cells treated with MEDI3622 alone, however, did not undergo a significant increase in proliferation (**Figure 1A**). The same effects on NK cell proliferation were observed when using PBMCs (**Figure 1B**). Moreover, ADAM17 blockade increased the sensitivity of NK cells to IL-15 for proliferation (**Supplementary Figure 1A**).

**Figure 2:**
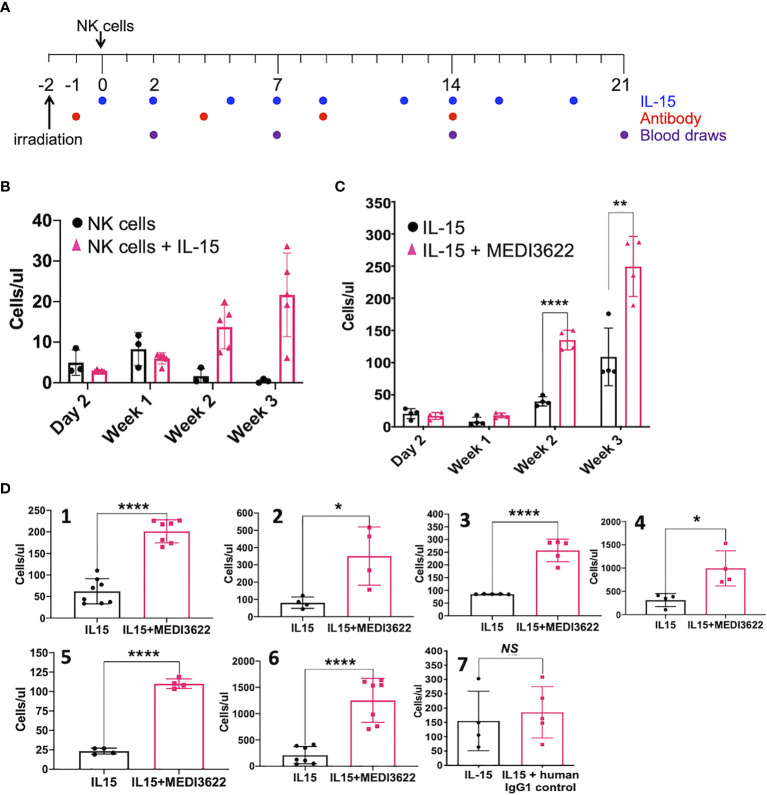
ADAM17 blockade enhances human NK cell proliferation by IL-15 *in vivo.* **(A)** Animal treatment schema. NSG mice were treated as described in the Methods. **(B)** Enriched NK cells were infused in the presence or absence of rhIL-15 (5μg), as indicated. Mouse peripheral blood was collected and the number of human CD45^+^ CD56^+^ CD3^−^ NK cells were enumerated by flow cytometry and are shown as cells/μl. Data are mean ± SD (n = 3 to 5 mice per group). **(C)** Additional mice were administered enriched NK cells from a separate donor plus rhIL-15 (5μg) ± MEDI3622 (10 mg/kg). Data are mean ± SD (n = 4 mice per group). **p < 0.01; ****p < 0.0001. **(D)** The experiment was performed as described in panel **(C)** NK cells were obtained from six separate donors (panels 1-6). Mice were also treated with rhIL-15 in the presence or absence of a human IgG1 control mAb (panel 7). Mouse peripheral blood was collected at day 21 following NK cell adoptive transfer and human CD45^+^ CD56^+^ CD3^−^ NK cells were enumerated by flow cytometry. Group data was tested for normality (Kolmogorov-Smirnov test) and the differences in means were calculated by comparing means ± SD using an unpaired two-sided Student’s t-test. n = 3 to 7 mice per group. *p < 0.05; **p < 0.01; ****p < 0.0001; ns, not significant.

We next evaluated the effects of blocking ADAM17 on NK cell expansion *in vivo*. Administration of human IL-15 or when expressed by a transgene stimulates the proliferation of transferred human NK cells in immunocompromised mice (9, 10, 12). As shown in the treatment schema in **Figure 2A**, NSG mice were administered enriched NK cells (4x10^6^) *iv*, rhIL-15 (5μg) *ip*, and/or MEDI3622 (10mg/kg) *ip*, and then circulating NK cells levels were monitored for 3 weeks. The adoptive transfer of NK cells in rhesus macaques revealed that these cells initially accumulated in the lung and by 24 hours they returned to the circulation (28). A similar process occurred for transferred human NK cells in immunocompromised mice (9). Therefore, we initially bled mice two days post-NK cell transfer to determine their baseline recirculating levels. Human NK cells (CD45^+^ CD56^+^ CD3^−^) were identified by a specific cell gating approach (**Supplementary Figure 1B**) and enumerated using cell counting beads. Baseline circulating levels of human NK cells were equivalent in mice treated with or without rhIL-15 (**Figure 2B**), whereas over time, their levels increased when in the presence of rhIL-15 (**Figure 2B**), as expected (10). In a separate experiment, baseline levels of circulating human NK cells from a different donor were again equivalent in mice treated with rhIL-15 in the presence or absence of MEDI3622, but by two weeks post-transfer, NK cell levels were significantly higher in the MEDI3622-treated mice (**Figure 2C**). The levels of NK cell expansion by IL-15 varied between donors (**Figures 2B, C**), and though the enhancement of this process by ADAM17 blockade was consistent, the levels of augmented proliferation also varied considerably between donors (**Figure 2D**, panels 1-6). Similar to the *in vitro* assays, the treatment of mice with control IgG did not enhance IL-15-mediated NK cell expansion (**Figure 2D**, panel 7), and treating mice with MEDI3622 alone did not induce NK cell expansion (**Supplementary Figure 1C**). D1(A12) is another function-blocking an anti-human ADAM17 mAb (29), and it also increased NK cell expansion by rhIL-15 (**Supplementary Figure 1D**). Collectively, our data reveal that ADAM17 induction in IL-15-stimulated NK cells reduces their proliferation potential.

### ADAM17 Regulates the Surface Density of CD62L on Proliferating NK Cells

**Figure 3:**
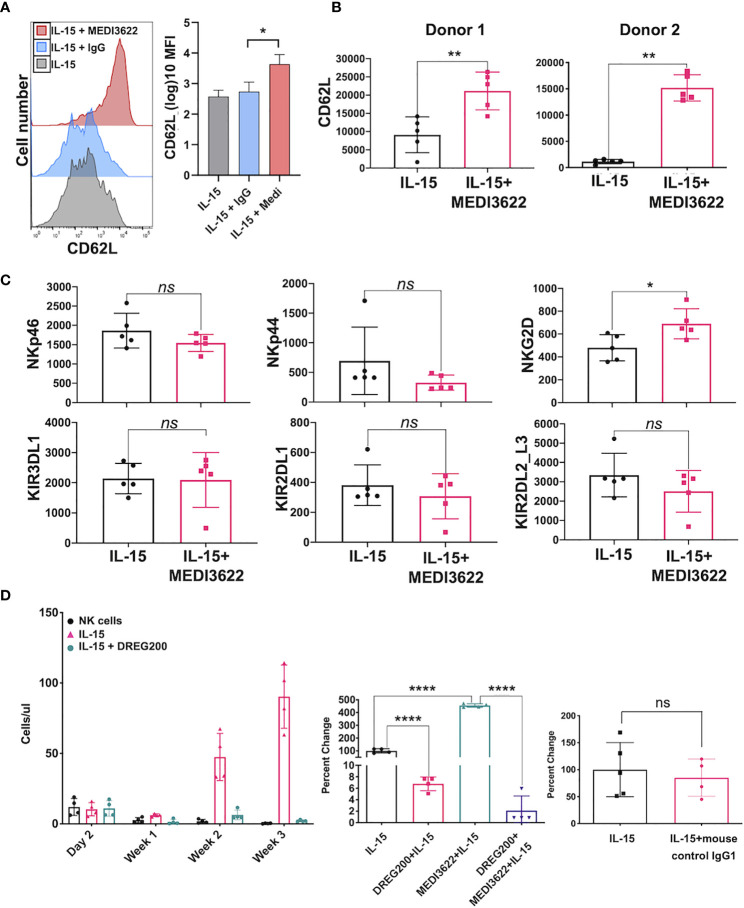
CD62L expression during NK cell proliferation and its role in their expansion in *vivo*. **(A)** NK cells were placed in culture for 7 days in the presence of rhIL-15 (10ng/ml) alone or in the presence of MEDI3622 (5 μg/ml) or an isotype-matched negative control mAb (IgG, 5 μg/ml). CD62L levels were determined by flow cytometry. The histograms show representative data and the bar graph shows mean ± SD of 3 independent experiments using leukocytes from separate donors. *p < 0.05. The y-axis on the bar graph indicates mean fluorescence intensity (MFI). Statistics were calculated as described in **Figure 1**. **(B)** Mice were administered enriched NK cells and rhIL-15 (5μg) in the presence or absence of MEDI3622 (10 mg/kg). After 3 weeks, mouse peripheral blood was collected, and relative CD62L expression levels were determined on human CD45^+^ CD56^+^ CD3^−^ NK cells by flow cytometry. Two separate experiments are shown using NK cells from different donors. The y-axis on the bar graphs indicates MFI. Data are mean ± SD (n = 5 mice per group). **p < 0.01. **(C)** The experiment was performed as described in panel B and various cell surface markers were evaluated. Data are representative of two separate experiments using NK cells from different donors. Data are means ± SD (n = 5 mice per group). *p < 0.05; ns = not significant. Data was analyzed by using unpaired two-tailed student’s t-test. **(D)** Mice were administered NK cells and rhIL-15 (5μg) in the presence or absence of DREG200 (10mg/kg) (left panel), DREG200 and/or MEDI3622 (10mg/kg) (middle panel), or mouse IgG isotype-matched mAb (10mg/kg) as a control for DREG200 (right panel). The number of NK cells in the peripheral blood were enumerated by flow cytometry and are shown as cells/μl or as percent change normalized to NK cells plus rhIL-15. Data are means ± SD (n = 4 to 5 per group). ****p < 0.0001; ns, not significant. Data was analyzed by using unpaired two-tailed student’s t-test.

ADAM17 has a broad array of substrates expressed by diverse cell types (15–17), and a small number of these are expressed by human NK cells (18). One very well described substrate of ADAM17 is CD62L (L-selectin), which is a “homing receptor” that directs most leukocytes from the blood into various tissue locations (30). Essentially all CD56^bright^ NK cells and a subset of CD56^dim^ NK cells in the peripheral blood express CD62L (31), and it undergoes a rapid downregulation in expression following IL-15 stimulation (32). This process was greatly reduced by MEDI3622 treatment (**Supplementary Figure 1E**). MEDI3622 treatment also resulted in markedly higher levels of CD62L on proliferating NK cells stimulated with IL-15 (**Figure 3A**). This was not entirely expected since prolonged stimulation of T cells has been shown to induce CD62L downregulation mainly by reduced gene transcription (33). ADAM17 also regulated the cell surface density of CD62L on NK cells *in vivo* during their proliferation by IL-15. NSG mice were treated as illustrated in **Figure 2A**, and at three weeks post-NK cell transfer, CD62L levels were evaluated on the circulating NK cells. In the presence of rhIL-15 and MEDI3622, NK cells had significantly higher levels of CD62L than did NK cells from mice treated with rhIL-15 alone (**Figure 3B**). We observed that the expression levels of various other cell surface determinants on proliferating NK were not affected by MEDI3622 treatment, including a sample of inhibitory and activating receptors (**Figure 3C**), though NKG2D expression was modestly increased (**Figure 3C**). The mechanism for this is unclear at this time and we are not aware of any studies showing that NKG2D is a substrate of ADAM17. Taken together, our findings reveal that CD62L is expressed at high levels during NK cell proliferation induced by IL-15, but undergoes considerable shedding by continuous ADAM17 activation.

### CD62L Is Required for NK Cell Expansion *In Vivo*

CD62L is involved in the migration of NK cells into lymphoid and peripheral tissues (34–36). The expansion of transferred human NK cells in immunocompromised mice occurs at various locations, including the spleen, liver, and bone marrow (9, 10, 12). We examined the contribution of CD62L to human NK cell proliferation *in vivo*. NSG mice were administered human NK cells alone or NK cells plus rhIL-15 in the presence or absence of DREG200, a well-described anti-human function-blocking mAb previously used in *in vivo* studies (37–39). Baseline levels of circulating NK cells were equivalent in all groups (**Figure 3D**, left panel). In contrast to mice that received NK cells and rhIL-15, very little NK cell expansion was observed in the presence of rhIL-15 and DREG200 (**Figure 3D**, left panel). Moreover, the enhanced expansion of NK cell that occurred in the presence of rhIL-15 and MEDI3622 was also blocked by DREG200 (**Figure 3D**, middle panel). Administration of control IgG instead of DREG200 did not affect NK cell proliferation by rhIL-15 (**Figure 3D**, right panel). These findings reveal that CD62L is critical for IL-15-mediated NK cell expansion *in vivo*, and thus its shedding by ADAM17 would likely impair their accumulation and/or stimulation in expansion niches. However, ADAM17 has numerous substrates that regulate cell activation and cell-cell interactions (17), and therefore substrates in addition to CD62L may also have a role in NK cell expansion. Indeed, we found that blocking CD62L by DREG200 did not affect IL-15-mediated NK cell proliferation *in vitro* (**Figure 1B**).

## Discussion

NK cells respond to various ligands and attached antibodies on tumor cells, resulting in natural cytotoxicity and antibody-dependent cell-mediate cytotoxicity (ADCC), respectively. NK cells also release several anti-tumor cytokines and chemokines that modulate other leukocyte subsets of the innate and adaptive immune system (1). Due to their assorted anti-tumor activities, adoptive NK cell therapies are being examined in a number of clinical trials (4). NK cells, however, tend to be shorter-lived cells following adoptive transfer and so cytokine stimulation is being examined to promote their expansion and survival (6, 7). Current strategies are focused on IL-15 and related agonists, which induce a potent proliferative signal for NK cells (13). Results from our study show that IL-15 activates ADAM17 upon short-term and prolonged stimulation of human NK cells. We show that blocking this sheddase resulted in a significant increase in NK cell proliferation both *in vitro* and *in vivo*. These findings thus suggest that ADAM17 activity during extended NK cell stimulation functions like a feedback system to modulate their proliferation, presumably through the cleavage of one or more critical substrates.

CD62L is a well characterized substrate of ADAM17 on leukocytes (16, 30), including NK cells (22, 23). CD62L is expressed by CD56^bright^ NK cells and a subset of CD56^dim^ NK cells (31), and of interest is that both NK cell subsets have been reported to be capable of high proliferation following cytokine stimulation (40). We show that cell surface levels of CD62L were markedly higher on proliferating NK cells *in vitro* and *in vivo* when blocking ADAM17. Childs and Berg have reported that *ex vivo* human NK cell proliferation in medium containing IL-15 with nicotinamide resulted in increased CD62L expression and NK cell expansion in immunodeficient mice (41). To directly examine whether CD62L may have a role in the *in vivo* expansion of human NK cells by IL-15, we blocked its function in a xenograft mouse model. This resulted in a dramatic reduction in NK cell expansion in the absence as well as presence of an ADAM17 function-blocking mAb. The expansion of transferred human NK cells in immunocompromised mice occurs at various locations (9, 10, 12). At this time, it has not been determined whether CD62L might direct NK cells to various expansion niches or primarily to one location, where upon initial proliferation these cells then traffic to other expansion niches. Blocking ADAM17 and CD62L shedding in other innate leukocytes, such as neutrophils, during sterile inflammation and infection also enhanced their migration to tissue locations (42, 43). Higher CD62L expression levels thus represent a potential underlying mechanism accounting for the increased expansion of NK cells upon blocking ADAM17. Indeed, others have reported that relatively small changes in L-selectin density can have significant effects on leukocyte migration (44, 45).

ADAM17 has numerous substrates and several regulate leukocyte activation (16, 17). Therefore, substrates in addition to CD62L may also play a role in NK cell expansion. Interestingly, we found blocking CD62L did not affect IL-15-mediated NK cell proliferation *in vitro*. Further studies will be required to identify additional mechanisms, direct and/or indirect, by which ADAM17 activation modulates NK cell proliferation. One candidate is IL-15Rα, a component of the trimeric receptor complex that binds IL-15, which has been reported to be cleaved by ADAM17 (46). Abrogation of its shedding could increase IL-15 presentation to IL-2/IL-15Rβ/γc on NK cells and enhance their stimulation and expansion.

Continuous IL-15 stimulation of NK cells has been reported to induce exhaustion (47). Thus, it will be important to assess the effects of ADAM17 blockade on the functional state of IL-15-stimulated NK cells. During NK cell exhaustion, various activating receptors have been shown to undergo downregulation, including NKG2D (48). We observed that NKG2D expression was modestly increased on IL-15-expanded human NK cells in mice treated with MEDI3622, and that other activating and inhibitory receptors did not significantly change in expression. Clinical studies have shown IL-15 administration to also have dose-limiting toxicities (13). Blocking ADAM17 in combination with IL-15 administration might allow for the administration of lower levels of the cytokine or its agonists and still achieve efficacious NK cell expansion, but with less toxicity or NK cell exhaustion. In support of this, we found that the treatment of NK cells with MEDI3622 increased their sensitivity to IL-15. Blocking ADAM17 may have additional anti-tumor effects as well. We have shown that ADAM17 inhibition increased NK cell ADCC and their production of INFγ (22, 49). Blocking ADAM17 activity in tumor cells can also enhance NK cell cytotoxicity. For instance, MHC class I-related chain molecules A and B (MICA and MICB) and natural cytotoxicity triggering receptor 3 ligand 1 (NR3LG1), also referred to B7-H6, are widely expressed by tumor cells (50, 51), and they have been reported to be substrates of ADAM17 (52–55). MICA/B and NR3LG1 are ligands of the NK cell activating receptor NKG2D and NKp30, respectively, and blocking their shedding increased tumor cell killing by NK cells (55, 56). The above findings reveal that ADAM17’s impact on NK cells is diverse (e.g., effector functions, proliferation, and trafficking) and multifactorial, thus addressing the effects of its inhibition on their function, especially *in vivo*, is complex.

In summary, our data demonstrates that rapid and prolonged induction of ADAM17 activity occurs in human NK cells stimulated by IL-15 and that this can limit their proliferation. CD62L is shown to play a role in IL-15-mediated NK cell expansion *in vivo* and its shedding by ADAM17 represents a potential underlying mechanism by which the sheddase regulates NK cell proliferation. A potential impact of these studies is that blocking ADAM17 may provide a therapeutic approach to increase NK cell proliferation by IL-15 and their anti-tumor function in patients.

## Data Availability Statement

The raw data supporting the conclusions of this article will be made available by the authors, without undue reservation.

## Ethics Statement

The animal study was reviewed and approved by Institutional Animal Care and Use Committee at the University of Minnesota.

## Author Contributions

HM, KD, and BW collected, assembled, analyzed and interpreted the data, and wrote the manuscript. NP contributed vital reagents. MF and JM analyzed and interpreted the data. All authors contributed to the article and approved the submitted version.

## Funding

This work was supported by grants from the NIH, award number R01CA203348. KD is a Lymphoma Research Foundation Grantee.

## Conflict of Interest

Author NP was employed by company AstraZeneca.

The remaining authors declare that the research was conducted in the absence of any commercial or financial relationships that could be construed as a potential conflict of interest.
