# Supplementary material for: Activation of ADAM17 by IL-15 Limits Human NK Cell Proliferation
Source: Front Immunol. 2021 Jul 22;12:711621. doi: 10.3389/fimmu.2021.711621 (PMC8339566; doi:10.3389/fimmu.2021.711621)
Supplement: Supplementary file 1 [file DataSheet_1.docx]

Supplementary Material

**
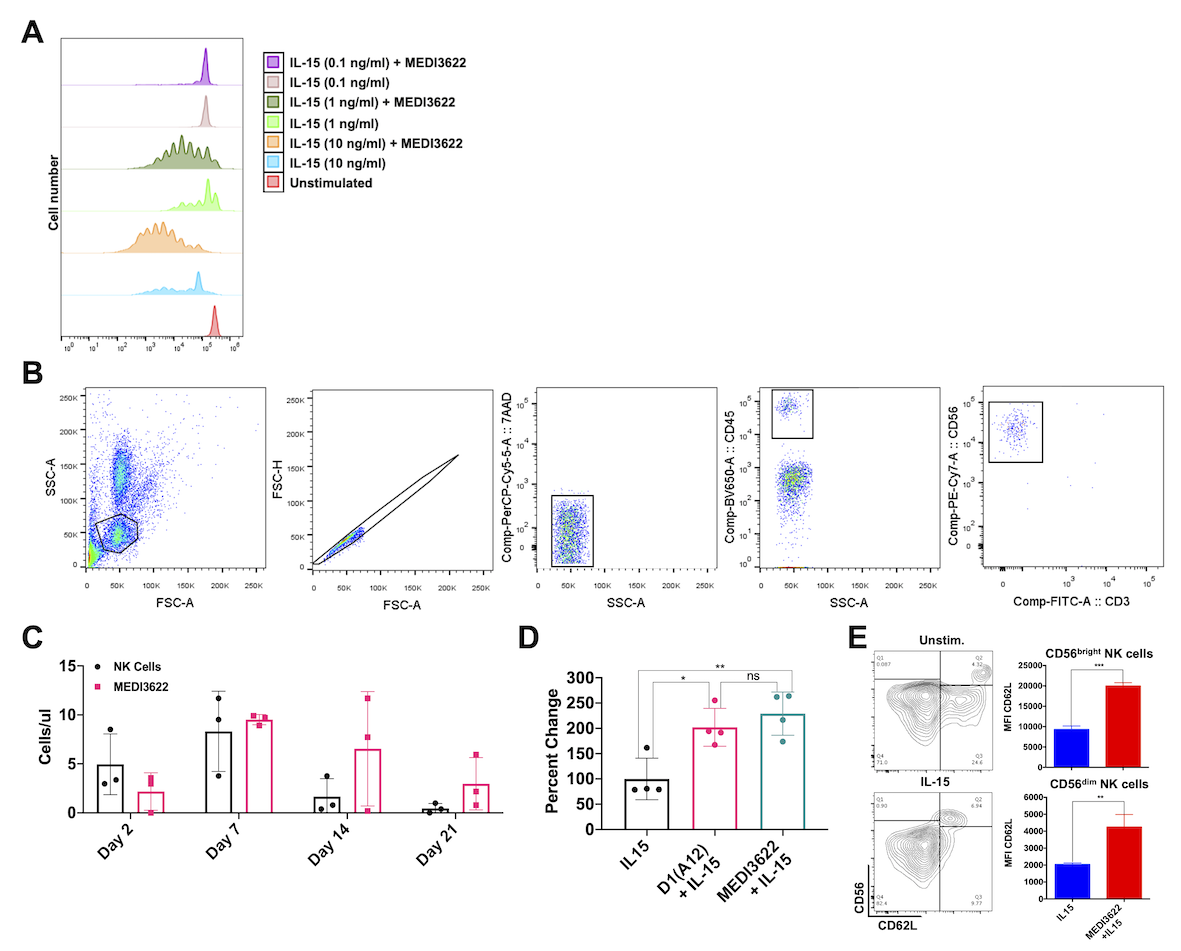
**

**Supplementary Figure 1.** (A) Human PBMCs were labeled with CellTrace Violet dye and placed in culture for 7 days with varying concentrations of rhIL-15 in the presence or absence of MEDI3622 (5μg/ml), as indicated. Cells were then harvested, and the NK cells were examined for CellTrace dye dilution by flow cytometry. Data are representative of 3 independent experiments using leukocytes from separate donors. (B) Flow cytometry gating strategy to enumerate adoptively transferred human NK cells, as described in the Methods. (C) Mice were administered NK cells in the presence or absence of MEDI3622 (10 mg/kg), as indicated. Circulating NK cells were enumerated by flow cytometry and shown as cells/μl. Data are means ± SD (n=3 mice per group). (D) Mice were administered NK cells and rhIL-15 in the presence or absence of MEDI3622 or D1(A12). After 3 weeks, the number of NK cells were enumerated by flow cytometry and are shown as percent change normalized to NK cells plus rhIL-15. Data are means ± SD (n=4 mice per group). Statistical significance is indicated as *p < 0.05; **p < 0.01; ns = not significant. (E) NK cells were placed in culture overnight in the presence or absence of rhIL-15 (10ng/ml), as shown in the contour plots, or rhIL-15 in the presence or absence of MEDI3622 (5 μg/ml), as shown in the bar graphs. Cell surface levels of CD62L were determined by flow cytometry. The contour plots show representative data and the bar graphs show mean ± SD of 3 independent experiments using leukocytes from separate donors. **p < 0.01; ***p < 0.001. Statistical analyses were performed as described in Figures 1 and 2.
